# Coupling and regulation mechanisms of the flavin-dependent halogenase PyrH observed by infrared difference spectroscopy

**DOI:** 10.1016/j.jbc.2024.107210

**Published:** 2024-03-20

**Authors:** Lea Schroeder, Niklas Diepold, Simon Gäfe, Hartmut H. Niemann, Tilman Kottke

**Affiliations:** 1 Biophysical Chemistry and Diagnostics, Department of Chemistry, Bielefeld University, Bielefeld, Germany; 2 Biophysical Chemistry and Diagnostics, Medical School OWL, Bielefeld University, Bielefeld, Germany; 3 Structural Biochemistry, Department of Chemistry, Bielefeld University, Bielefeld, Germany

**Keywords:** allosteric regulation, bacterial metabolism, enzyme mechanism, flavin adenine dinucleotide (FAD), flavoprotein, fourier transform IR (FTIR), halogenase, monooxygenase, protein conformation, tryptophan

## Abstract

Flavin-dependent halogenases are central enzymes in the production of halogenated secondary metabolites in various organisms and they constitute highly promising biocatalysts for regioselective halogenation. The mechanism of these monooxygenases includes formation of hypohalous acid from a reaction of fully reduced flavin with oxygen and halide. The hypohalous acid then diffuses via a tunnel to the substrate-binding site for halogenation of tryptophan and other substrates. Oxidized flavin needs to be reduced for regeneration of the enzyme, which can be performed *in vitro* by a photoreduction with blue light. Here, we employed this photoreduction to study characteristic structural changes associated with the transition from oxidized to fully reduced flavin in PyrH from *Streptomyces rugosporus* as a model for tryptophan-5-halogenases. The effect of the presence of bromide and chloride or the absence of any halides on the UV-vis spectrum of the enzyme demonstrated a halide-dependent structure of the flavin-binding pocket. Light-induced FTIR difference spectroscopy was applied and the signals assigned by selective isotope labeling of the protein moiety. The identified structural changes in α-helix and β-sheet elements were strongly dependent on the presence of bromide, chloride, the substrate tryptophan, and the product 5-chloro-tryptophan, respectively. We identified a clear allosteric coupling in solution at ambient conditions between cofactor-binding site and substrate-binding site that is active in both directions, despite their separation by a tunnel. We suggest that this coupling constitutes a fine-tuned mechanism for the promotion of the enzymatic reaction of flavin-dependent halogenases in dependence of halide and substrate availability.

Halogenated metabolites are widespread in nature with a variety of biological functions (1). Besides the central role that iodinated thyroid hormones play in humans, 4-chloroindole-3-acetic acid is a member of the auxin family of hormones directing plant growth and morphology (2). Bacteria produce halogenated pigments such as the brominated xanthomonadin for prevention of photodamage (3). Recently, a brominated neurotoxin identified in cyanobacteria was found to be the cause for a severe intoxication of water birds and birds of prey (4). To better understand how these compounds are synthesized in nature, there has been an ongoing effort to find the enzymes responsible for the halogenation and to understand their reaction mechanisms.

Flavin-dependent halogenases are an important halogenase class for the halogenation of metabolites with a variety of biological activities such as the antibiotic chloramphenicol, the antitumor agent rebeccamycin, or the vasorelaxant malbrancheamide (5, 6, 7, 8, 9). Moreover, halogenases have a great potential for biocatalytic application in the synthesis of halogenated fine chemicals. In contrast to chemical routes, enzymatic halogenation proceeds with high regioselectivity under mild conditions (5, 10, 11). Finally, halogenases can be used in fermentative processes (12).

PyrH from *Streptomyces rugosporus* is a model enzyme for halogenation at the 5-position of tryptophan and tryptophan-containing peptides (13, 14, 15, 16, 17, 18). Crystal structures of PyrH have been solved showing the typical arrangement of a box-shaped binding module for the cofactor FAD and a triangular pyramid covering the substrate-binding pocket in between (Fig. 1*A*) (19). According to the currently accepted mechanism, fully reduced FAD (FADH^−^) reacts with molecular oxygen and halide to form hypohalous acid (20). A tunnel connects the FAD-binding pocket with the substrate located in a distance of 10 Å (20, 21). In the substrate-binding site, the halogenating agent reacts with tryptophan to form the halogenated product. FADH^−^ is then regenerated from oxidized FAD (FAD_ox_) by a flavin reductase under consumption of NADH. Recently, it has been demonstrated that FAD_ox_ bound to PyrH can be photoreduced in the presence of a sacrificial reductant (Fig. 1*B*) (22). This photoreduction has been successfully employed to drive biocatalysis for full conversion of tryptophan to 5-chloro-tryptophan. The light-driven reaction proceeds without any auxiliary enzymes such as a flavin reductase, alcohol dehydrogenase, or catalase. Apart from these advantages for synthesis, the photoreduction paves the way to investigate crucial aspects of the halogenase mechanism by spectroscopy because the conversion can be controlled by light.

**Figure 1:**
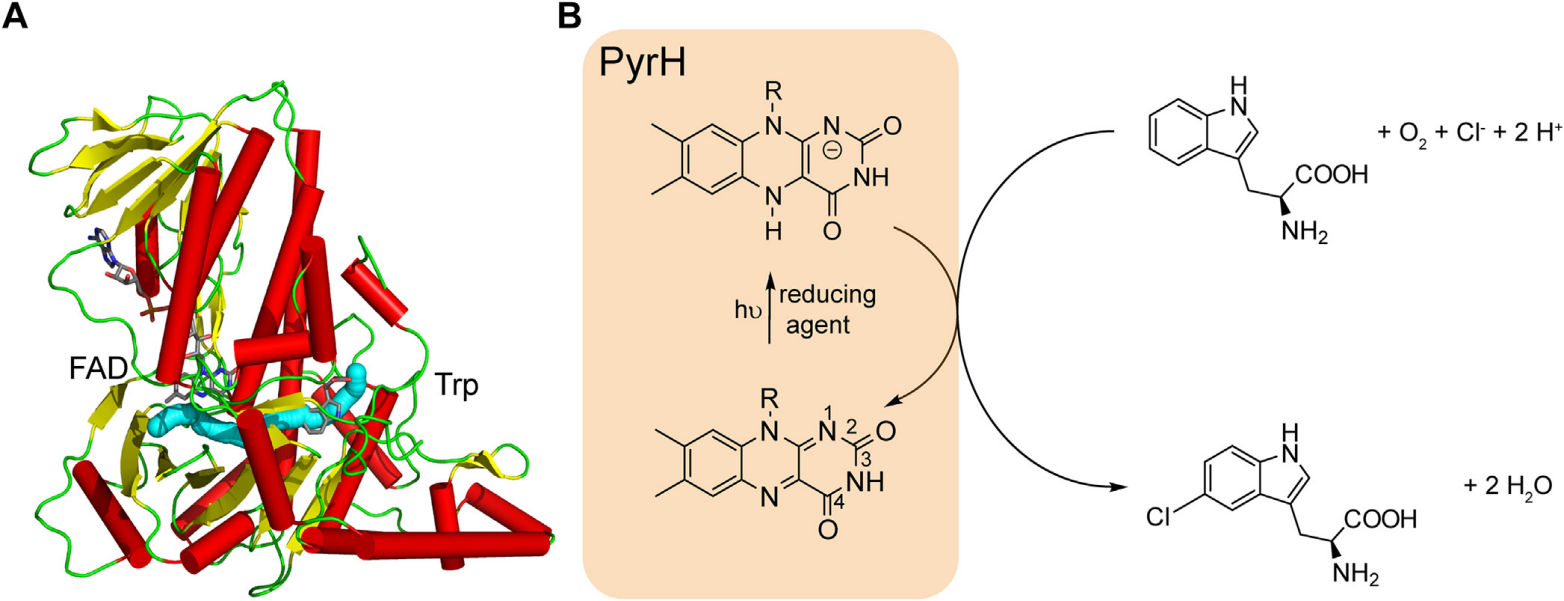
**Structure of PyrH and substrate conversion by the halogenase.***A*, structure of the halogenase PyrH with cofactor FAD and substrate tryptophan (PDB entry 2WET). Helices are shown in *red*, β-sheets in *yellow*, and loop structural elements in *green*. The tunnel for HOX diffusion from cofactor to substrate is highlighted in *blue* (57). *B*, reaction scheme for the halogenation of tryptophan by the halogenase PyrH. To drive the catalysis, the photoreduction of FAD_ox_ to FADH^−^ has been established instead of the cellular reduction of FAD_ox_ by NADH, which is mediated by a flavin reductase. The numbering of the flavin ring is indicated. The ribityl phosphate and adenosine monophosphate moieties of FAD are represented by R. FADH^−^, fully reduced FAD; FAD_ox_, oxidized FAD.

PyrH shows a strong preference for the conversion of chloride but will accept bromide in the absence of the former (13). Such a preference has been observed for several tryptophan halogenases, but a preference for bromide has been found in the halogenases from marine organisms like BrvH and KrmI (23, 24). Also, dibromination of their substrates was observed for some halogenases (25). Even a preferred iodination has been suggested for VirX1 (26) but later attributed to a conversion with hypoiodous acid in solution (27). Results from stopped-flow kinetic analysis point to a higher reactivity of bromide than chloride with the intermediate flavin hydroperoxide, which would favor bromination over chlorination in general (28). Another influence might be the steric preference in the substrate-binding site for a specific hypohalous acid (28). Accordingly, the selection mechanism for the halide remains unclear and is of general importance for our understanding of enzymatic halogenation.

The binding pockets of FAD and tryptophan are separated from each other by a distance of around 10 Å (20). Accordingly, the reduction of FAD_ox_ and formation of the hypohalous acid might proceed independently of the binding and conversion of tryptophan (29). An allosteric coupling mechanism has been proposed based on the analysis of crystal structures of the tryptophan 7-halogenase PrnA (20). Reduction of FAD_ox_ to FADH^−^ in the crystal led to an increase in disorder at the substrate-binding site, whereas binding of tryptophan increased disorder close to FAD. Moreover, it has been found in several crystal structures of the tryptophan 6-halogenase Thal that binding of tryptophan is associated with a loss in electron density for FAD (30). To our knowledge, spectroscopic characterization of this coupling has not been performed to date.

FTIR difference spectroscopy (31, 32) has been successfully used to elucidate the function and mechanism of numerous enzymes but has not been applied to halogenases. The difference approach is particularly suitable for the study of light-triggered processes because the reaction then proceeds without any change in sample concentration or buffer composition. Accordingly, flavin reactions in photoreceptors and photoenzymes have been studied in detail (33, 34, 35, 36). Both changes in structure of the cofactors and of the protein moiety can be detected simultaneously. Restructuring of secondary structural elements are revealed by shifts in the vibrational frequency of characteristic amide I (at 1695–1615 cm^−1^) and amide II (at 1570–1520 cm^−1^) modes of the protein backbone (37). Typical vibrations of the cofactor flavin in the oxidized and fully reduced state have been assigned in solution and bound to enzymes (38, 39, 40, 41).

Here, we use the photoreduction of FAD_ox_ bound to PyrH to investigate the mechanism of halogenases. We show first evidence for a halide-specific selection mechanism that results in changes in secondary structure of the protein. An allosteric feedback mechanism between the substrate and FAD-binding site via a loop has been proposed in literature based on the analysis of crystal structures (20, 30). We demonstrate that this mechanism exists in solution at ambient conditions and show a structural contribution by β-sheet rearrangements.

## Results

### Redox potential of PyrH

We have demonstrated that the halogenase PyrH can be prepared to bind oxidized FAD with an occupancy of up to 70% after reconstitution (22), which facilitates the investigation of the properties and mechanism of the enzyme. First, we analyzed the redox potential of FAD in PyrH by employing an established approach using xanthine/xanthine oxidase (42, 43) to slowly increase the potential and 2-antraquinone-sulfonate as an indicator dye. Both the absorption changes of the dye and of FAD were dissected simultaneously using the full spectral range to obtain the conversion with respect to the redox potential and to generate a Nernst plot (Fig. S1). The redox potential for PyrH was determined to be *E* = −248 (±4) mV *versus* standard hydrogen electrode at pH 8 (Fig. S2), which is close to that of free FAD in solution of *E* = −230 mV *versus* standard hydrogen electrode at pH 8 (44).

### FTIR difference spectrum and band assignment

**Figure 2:**
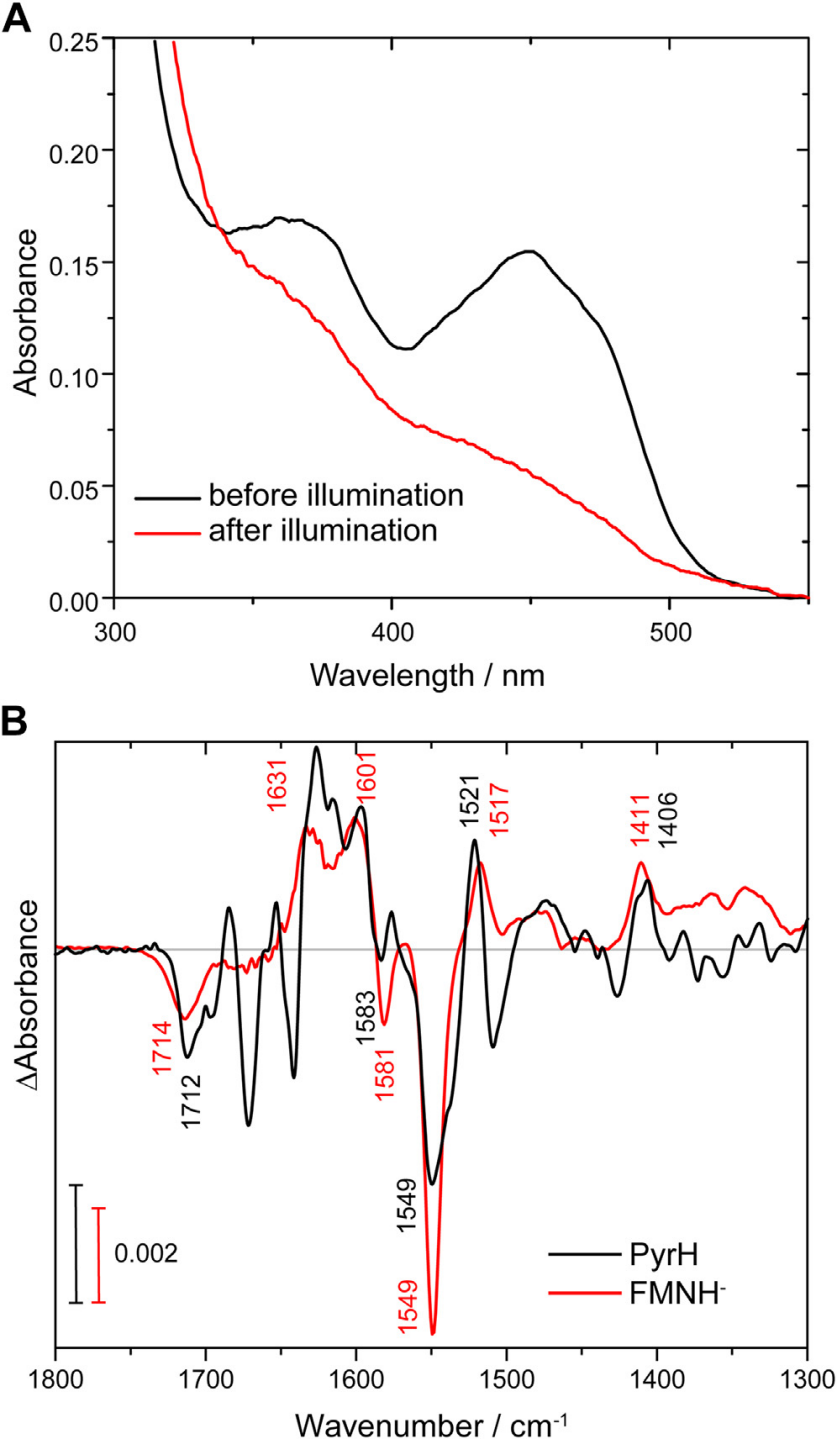
**Photoreduction of FAD**_**ox**_**in PyrH.***A*, UV-vis spectra of PyrH in the presence of DTT before and after illumination with *blue* light showing the formation of FADH^−^ from FAD_ox_ inside the halogenase. Radical intermediates were not observed. *B*, FTIR difference spectra of PyrH with bound FAD in the presence of DTT before and after illumination in comparison to the formation of fully reduced FMN (FMNH^−^) from FMN in solution (taken from (41)). Negative bands correspond to the oxidized, positive bands to the fully reduced state. Characteristic signals for the fully reduced state at 1411/1406 cm^−1^ and at 1521/1517 cm^−1^ confirm the formation of FADH^−^ in PyrH. FADH^−^, fully reduced FAD; FAD_ox_, oxidized FAD.

The FAD bound to the halogenase PyrH can be fully reduced by an external reducing agent such as EDTA if coupled with blue light illumination (22). For FTIR difference spectroscopy, we used DTT instead of EDTA because it does not form gaseous products and contributes negligible signals to the investigated spectral window upon oxidation. A complete conversion of the sample to the fully reduced state in the presence of DTT was obtained in a similar fashion as with EDTA, as demonstrated by UV-vis spectroscopy (Fig. 2*A*). The characteristic absorption band of FAD_ox_ in PyrH at 449 nm is lost upon reduction and replaced by the broad absorbance of FADH^−^ (22, 45).

The procedure was applied to a concentrated protein solution of >1 mM for FTIR spectroscopy in the presence of 20 mM chloride and a difference signal was detected after several minutes of illumination. The long exposure time was attributed to a low quantum efficiency of the photoreduction. Moreover, FADH^−^ might react with some residual oxygen in the sample, which remained after incubation with 35 mM DTT for at least 2 h in a sealed cuvette. As a result of the incubation and illumination, the cuvette was rendered fully anaerobic and the enzymatic reaction stopped with the formation of FADH^−^. Under these anaerobic conditions, halogenation is not observed. Instead, the FTIR difference spectrum shows the conversion of FAD_ox_ to FADH^−^ (Fig. 2*B*) as identified by comparison to the difference spectrum of formation of the fully reduced FMN in solution (41). We observed the characteristic positive bands of the fully reduced flavin at 1406 cm^−1^ and 1521 cm^−1^ (38, 41) demonstrating that under FTIR spectroscopic conditions, the reaction proceeds similar as observed in the UV-vis spectral region (22).

**Figure 3:**
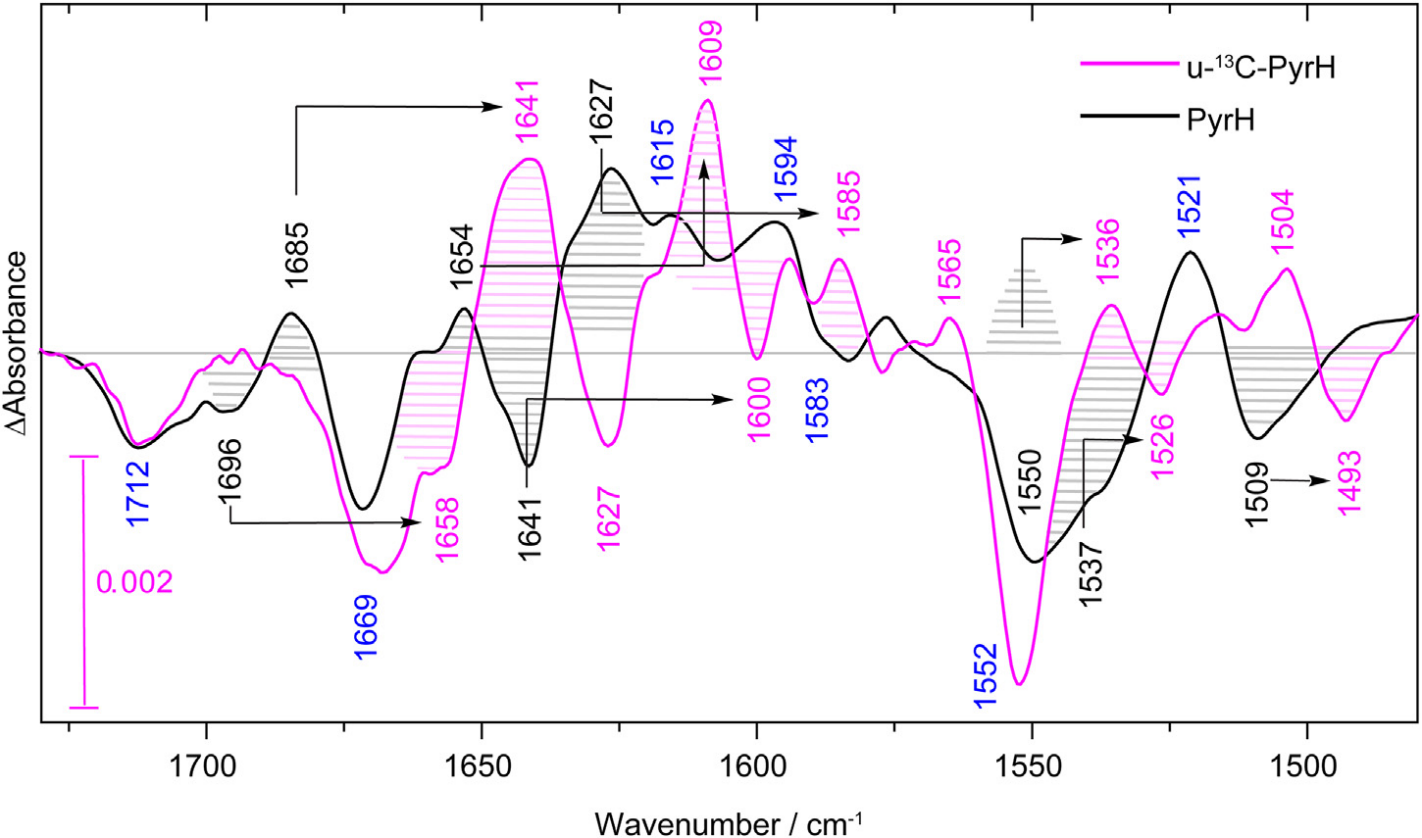
**Light-induced FTIR difference spectra of u-**^**13**^**C-labeled PyrH and PyrH with natural isotope abundance.** Both samples bound FAD at natural abundance of isotopes. Contributions from the protein moiety such as changes in secondary structure were identified by their characteristic downshifts of ∼40 cm^−1^ for amide I bands in the range of 1695 to 1615 cm^−1^ and ∼10 cm^−1^ for amide II bands in the range of 1570 to 1520 cm^−1^ (indicated by *arrows*). The frequencies of bands originating from FAD do not shift and are indicated in *blue*. The spectrum of u-^13^C-labeled PyrH was scaled to the unlabeled spectrum using the flavin band at 1712 cm^−1^.

A number of additional difference bands to those of the FAD reduction were observed. To separate contributions of FAD from those of the protein moiety, we aimed for selective ^13^C-isotope labeling of the protein moiety while keeping the FAD at natural abundance of isotopes. We expressed PyrH in minimal medium with u-^13^C_6_ glucose as the only carbon source. FAD was not bound after purification of the enzyme (Fig. S3) and the reconstitution proceeded with FAD at natural abundance. This procedure replaces the commonly used exchange of solvent to D_2_O as it has a superior labeling efficiency of 98% (46). Moreover, amide I bands of secondary structural elements are only slightly sensitive to the exchange in D_2_O (37), whereas they undergo a strong downshift of about 40 cm^−1^ upon ^13^C labeling. As a result, difference bands originating from the FAD cofactor do not shift in frequency upon labeling, whereas bands from the protein moiety undergo a ^13^C-dependent downshift (Fig. S4). Difference spectra were obtained of PyrH at natural abundance and of u-^13^C-PyrH with shifts by labeling as indicated (Fig. 3 and Fig. S5). Accordingly, additional positive bands of FADH^−^ were identified at 1615 cm^−1^ and 1594 cm^−1^, which we tentatively assign to C=O and C=C stretching vibrations, respectively. Negative bands of FAD_ox_ can be found at typical frequencies for oxidized flavins at 1712 cm^−1^, 1669 cm^−1^, 1583 cm^−1^, and 1552 cm^−1^ (for an overview see Table S1) (38, 40, 47).

All other remaining bands are assigned to the protein moiety, which are caused by changes in secondary structure and/or by changes in amino acid side chains (37). Changes in secondary structure are characterized by prominent signals with downshifts by ^13^C-labeling of the amide I and amide II bands of about 40 cm^−1^ and 10 cm^−1^, respectively. Broad negative bands at around 1700 cm^−1^ and 1550 cm^−1^ accordingly contain not only contributions from FAD_ox_ but also from secondary structure because both bands become sharper and more uniform upon labeling. The band at 1552 (−) cm^−1^ increases in strength upon labeling, which indicates a positive contribution of the protein moiety at natural abundance at 1550 (−) cm^−1^ that partly compensates the strong FAD signal and then shifts to 1536 (+) cm^−1^ upon labeling. Prominent signals in the amide I spectral region at 1696 (−) cm^−1^, 1685 (+) cm^−1^, 1654 (+) cm^−1^, 1641 (−) cm^−1^, and 1627 (+) cm^−1^ downshift upon labeling. These signals are accompanied by corresponding signals in the amide II region at ∼1550 cm^−1^, ∼1537 cm^−1^, and 1509 cm^−1^. The latter signal is outside the typical amide II range but in agreement with assignments on other proteins (48). Furthermore, each band shows an unusual intensity for changes in single amino acids either with or without labeling, corroborating their assignment to changes in secondary structure (37). Accordingly, changes in α-helical and β-sheet structure were detected with characteristic frequencies at 1654/1641 cm^−1^ and 1685/1627 cm^−1^, respectively. The other change at 1696 cm^−1^ requires more information for an assignment. All these signals might reflect an activation mechanism of the protein in response to favorable reaction conditions for turnover, that is, the presence of FADH^−^.

### Effect of halide binding studied by UV-vis and FTIR spectroscopy

**Figure 4:**
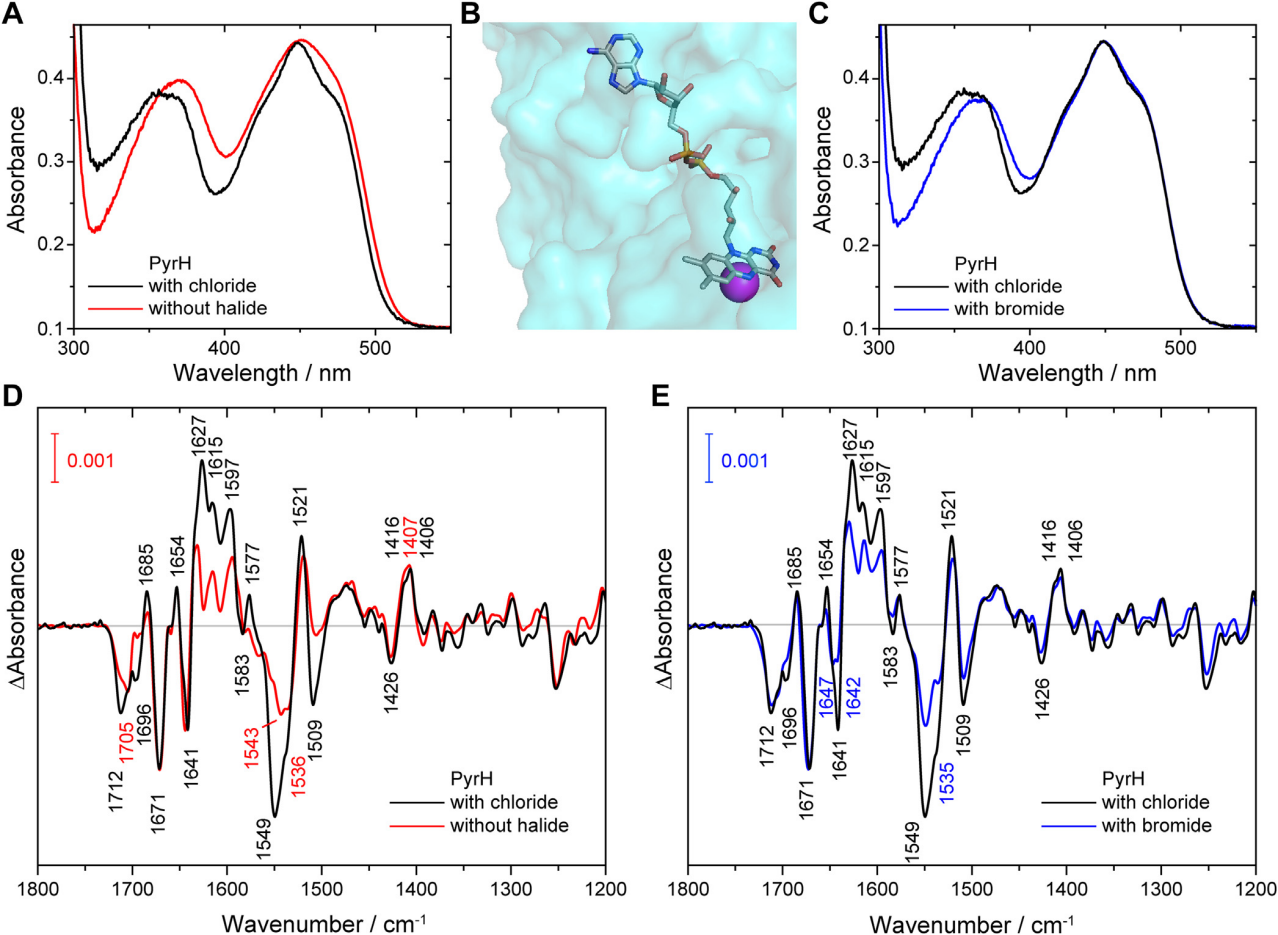
**Dependence of PyrH spectra on the presence of halide.***A*, UV-vis spectra of FAD bound to PyrH in the presence of chloride and in the absence of halide demonstrate a direct influence of the charged halide on the FAD-binding pocket. *B*, structure of the binding pocket of FAD with chloride bound in the halogenase PyrH (PDB entry 2WET). *C*, UV-vis spectra of FAD bound to PyrH in the presence of bromide and chloride, respectively. *D*, FTIR difference spectrum of the photoreduction of FAD_ox_ to FADH^−^ in PyrH in the presence of chloride in comparison to that without any halide. *E*, FTIR difference spectrum in the presence of bromide in comparison to that with chloride. Stronger changes in secondary structure in the presence of chloride were identified at around 1620 (+) cm^−1^ and at 1509 (−) cm^−1^ as compared to the sample with bromide and to the sample without halide. Moreover, differences in the C_4_=O band at around 1700 (−) cm^−1^ demonstrate that the local surrounding of FAD differs depending on the availability of the halide. Spectra without halide and with bromide were scaled to spectrum with chloride to the band at 1406 cm^−1^. FADH^−^, fully reduced FAD; FAD_ox_, oxidized FAD.

To investigate the regulation mechanisms of PyrH in response to the presence and the absence of substrates and products, we varied the composition of the sample. As chloride is the preferred halide for PyrH, samples without any halide and with 20 mM bromide were obtained by using the respective chloride-free buffer already for the washing and reconstitution steps. The obtained samples were analyzed using UV-vis spectroscopy and showed significant differences depending on the presence and the absence of chloride and bromide (Fig. 4, *A*–*C*). It should be noted that FAD remained bound to PyrH in all three samples after extensive washing steps and the FAD occupancy was determined to around 60%. FAD in solution shows two absorption maxima at around 380 nm and 450 nm (49, 50). Upon binding to the protein, both maxima develop a fine structure and the maximum at 380 nm downshifts. The extent of these changes varies depending on the polarity and H-bonding environment of the flavin-binding pocket (51). Stronger downshift and fine structure are associated with more localized H-bonding and less polarity. A looser binding would therefore mimic more closely the spectrum of FAD in solution. Comparing the spectra of the halogenase in the presence and the absence of chloride, we see that the band at 380 nm shifts to lower wavelength in the presence of chloride and the band at 450 nm shows a clear fine structure (Fig. 4*A*). The spectrum of the sample in the absence of chloride closely resembles that of free FAD in solution albeit being bound to the protein. The crystal structure of the FAD-binding site of PyrH shows a chloride, which is located right next to the central ring of the isoalloxazine (Fig. 4*B*) (19). If this binding site was empty or filled with water instead, the binding pocket of the FAD might become more flexible and more polar resulting in less defined interactions and less coupling with the protein moiety. Such enhanced flexibility and polarity might explain the observed difference in the UV-vis spectra. The spectrum in the presence of bromide shows a fine structure at 450 nm identical to that in the presence of chloride. However, the 380 nm-band is red-shifted indicating a more polar environment than in the presence of chloride and a less ordered H-bonding structure (Fig. 4*C*). As all three samples show distinguishable UV-vis spectra, samples with no halide as well as samples with only bromide were prepared successfully. In conclusion, the UV-vis spectra might identify the preference of the halogenase for a halide because binding of chloride induces the most defined environment of FAD_ox_ in PyrH.

All three samples were further analyzed for changes in the FAD-binding pocket and in secondary structure upon photoreduction using FTIR difference spectroscopy. The influence of halide on the FAD that was observed using UV-vis spectroscopy can also be seen in the IR difference spectra (Fig. 4, *D* and *E*). We observed changes in the C_4_=O-band of FAD_ox_ at 1712 (−) cm^−1^. The band maximum downshifts to 1705 cm^−1^ without chloride caused by an increase in the number or strength of H-bonds to the C_4_=O of FAD that may be formed by the presence of water instead of chloride in the binding pocket. When bromide is present instead of chloride, the C_4_=O band remains at the same maximum confirming that bromide is bound in the same binding pocket as chloride. The C_4_=O-band is broader in the absence of halide from which we infer that different binding poses of the isoalloxazine ring are present, of which the halide preferentially stabilizes one pose. Further prominent differences between the spectra are caused by the extent of changes in secondary structure. In the absence of halide, changes seem to be vastly reduced as indicated by the reduction of signals at around 1627 (+) cm^−1^ and at 1509 (−) cm^−1^. The band at around 1550 cm^−1^ cannot be easily interpreted as it consists of positive as well as negative contributions by secondary structure and a prominent contribution by FAD_ox_. The observed changes in secondary structure show the same band maxima in the presence of chloride as well as bromide but appear smaller in extent in the latter case. Therefore, using IR difference spectroscopy, we show that the presence of chloride during the photoreduction of FAD allows for strong changes in secondary structure, whereas the absence of any halide leads to a strong reduction in response. Accordingly, some feedback exists for the halogenase to the presence of favorable reaction conditions.

### Effect of substrate and product binding studied by FTIR spectroscopy

**Figure 5:**
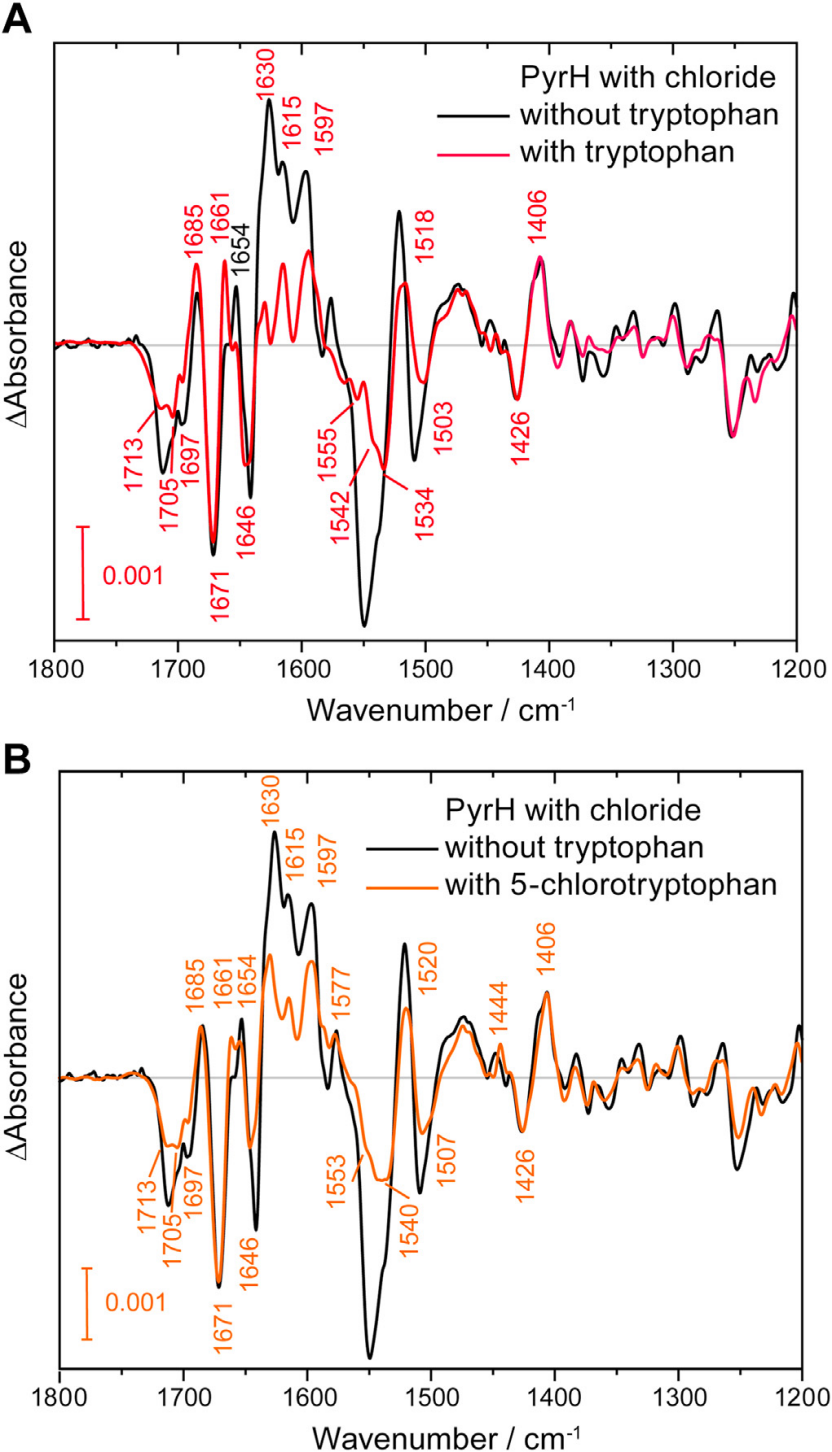
**FTIR difference spectrum of PyrH with chloride in the presence of the substrate tryptophan or the presence of the product 5-chlorotryptophan.** Changes in secondary structure depend strongly on the presence or the absence of substrate tryptophan (*A*) and product 5-chlorotryptophan (*B*), respectively. Moreover, changes in the FAD-binding pocket are observed in the presence of tryptophan compared to the absence of substrate, as visible at around 1700 (−) cm^−1^. Spectra were scaled to the band at 1406 cm^−^^1^.

In the next experiments, binding of tryptophan as the major substrate of the reaction was investigated for its influence on the protein structure and in particular on the cofactor-binding pocket. We investigated samples with 20 mM chloride in the presence of 3 mM of either the substrate tryptophan or the product 5-chlorotryptophan. FTIR difference spectra upon photoreduction showed significant differences when tryptophan or 5-chlorotryptophan are present compared to their absence (Fig. 5). Both spectra show a shift of the band at 1654 cm^−1^ to 1661 cm^−1^. Therefore, the appearance of a band at 1661 cm^−1^ can be regarded as a marker band for the occupancy of the tryptophan-binding pocket. While only one maximum occurs with tryptophan, 5-chlorotryptophan shows a second maximum remaining at 1654 cm^−1^. Interestingly, we see an influence of the tryptophan binding on the structure of the FAD-binding pocket by analyzing the C_4_=O band at >1700 cm^−1^. Tryptophan binding causes a change in the environment of FAD to form two major binding poses with two maxima instead of a single pose. Moreover, changes in secondary structure at around 1627 (+), 1550 (−), and 1509 (−) cm^−1^ are very small in the presence of tryptophan compared to other samples. The binding of 5-chlorotryptophan also leads to a reduction of secondary structure changes, albeit clearly smaller in extent. This difference could reflect the lower binding affinity of the product compared to the substrate, which is unlikely considering the high concentration of product in the FTIR experiments. In contrast, the high similarity in the band pattern at >1700 cm^−1^ speaks for a different effect of substrate and product on the extent of secondary structure changes upon FADH^−^ formation. The reduction in protein response strongly suggests in agreement with literature (19, 28) that major rearrangements in secondary structure are not required for the reaction to proceed after binding of substrate. We conclude that an allosteric feedback mechanism is present in solution, which not only reacts to the occupancy of the tryptophan-binding pocket but differentiates between the substrate and the product.

## Discussion

We demonstrated that light-induced FTIR difference spectroscopy can be applied to address open questions in the halogenation mechanism. Here, we investigated the response of the enzyme to the reduction of FAD_ox_ in the presence of the substrates chloride, bromide, and tryptophan as well as of the product 5-chlorotryptophan. By using selective isotope labeling of the protein moiety, we identified several changes in secondary structure especially in the α-helical and β-sheet spectral region that take place concomitant to the reduction of FAD_ox_. Of note, all observations were obtained by reduction of the FAD_ox_ but will qualitatively accompany the oxidation of FADH^−^ during the formation of HOCl with the opposite sign.

Some of these prominent structural changes induced by photoreduction of flavin are strongly impaired by bound tryptophan, which demonstrates the direct coupling of cofactor and substrate binding sites. One prominent signal is found at 1627 to 1630 (+) cm^−1^ characteristic for a change in β-sheet structural elements. The assignment is supported by the accompanying smaller signal at 1685 (+) cm^−1^, which is typically present for antiparallel β-sheets but not for parallel β-sheets (37). This change in β-sheet is limited to the absence of tryptophan and linked allosterically to flavin- and tryptophan-binding pockets (Figs. 4 and 5). Changes in β-sheets have not been described by crystal structure analysis previously. For PrnA, a comparison of structures with bound FAD_ox_ and FADH^−^ shows some increase in antiparallel β-sheet content by elongation of β18 (Fig. S6) (20). This increase might be indicative of larger rearrangements of the antiparallel β-sheet taking place in solution, as structural changes might be less pronounced in the crystal than in solution (52). There is only one alternative β-sheet conserved in flavin-dependent halogenases, which is parallel and distantly located from both binding sites. We propose that these β-sheet rearrangements are part of the allosteric network between flavin- and tryptophan-binding pocket in addition to previously suggested pathways: Dong *et al*. identified a direct communication between the two binding pockets via a loop at around G48 of PrnA close to the flavin (20). This loop undergoes strong rearrangements including a glutamate residue E49 and extends towards the tryptophan substrate.

Other observed changes in secondary structure are less affected by the presence of bound tryptophan. A prominent structural change is found from 1641(−) to 1654(+) cm^−1^ in the absence of tryptophan and from 1646(−) to 1661(+) cm^−1^ in the presence of tryptophan, which originates from a rearrangement in α−helical structure upon reduction of flavin. This signal pair might reflect the reorganization of the substrate-binding loop. For PyrH, both open and closed conformation of the substrate-binding loop form a partial α-helical structure, albeit involving different amino acids (Fig. S7) (19). It should be noted in this tentative assignment that PyrH is not fully homologous in structure to PrnA and Thal, in particular concerning the α-helical tryptophan-binding loop. The loop in PyrH is inserted at a different position in the sequence. In PrnA and Thal, the binding loop is only partially α-helical in the substrate-bound state, whereas it becomes dynamic in its open conformation (20, 30, 53). For PrnA, the substrate-binding loop loses the structure of α-helical elements capping the binding loop when FADH^−^ is bound, which provides evidence for the coupling of flavin- and substrate-binding sites (20). A structure of PyrH with bound FADH^−^ has not been determined yet for direct comparison with the FTIR experiments.

In addition, we analyzed the interactions of FAD_ox_ with the binding pocket in dependence of the presence of halide by FTIR and UV-vis spectroscopy. The absence of halide induces a downshift and broadening of the C_4_=O vibration of flavin and a loss of fine structure in the UV-vis spectrum, both indicating an access of water to the flavin moiety in the binding pocket and an increase in flexibility. The infrared band in the absence of halide remains distinct in linewidth and frequency (1705 cm^−1^) from that of flavin in water (at 1714 cm^−1^, see Fig. 2) which argues against a complete release of the isoalloxazine ring from the binding pocket as observed for Thal upon binding of tryptophan (30). However, the broadening of the C_4_=O band in the FTIR experiments upon binding of tryptophan supports the previous observation that isoalloxazine binding is destabilized. Of note, we see an effect of tryptophan binding on the flavin C_4_=O signal, which is direct evidence for an allosteric coupling from substrate to cofactor site. All observed changes are summarized in Table 1.

**Table 1:** Overview of the extent of changes in secondary structure observed in the photoreduction of FAD_ox_ in PyrH in dependence of the presence of halide, substrate, and product

Flavin-binding site	Substrate-binding site		
FAD_ox_ ↔ FADH^−^	Empty	Trp	5-Cl-Trp
Cl^−^	+++	+	++
Br^−^	++	n. d.	n. d.
No halide	+	n. d.	n. d.

n. d., not determined.

**Figure 6:**
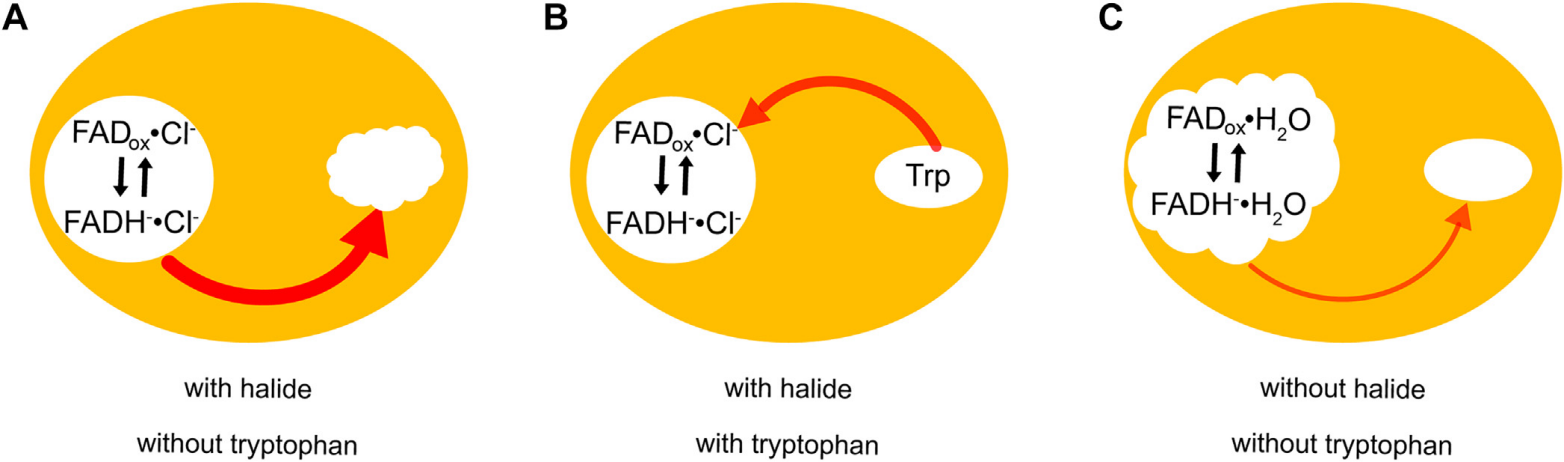
**Proposed coupling and regulation mechanisms in PyrH.** The strength of the arrows indicates the extent of the conformational change accompanying the reduction or oxidation of the cofactor. Such oxidation of the cofactor accompanies the formation of hypohalous acid. *Wavy circles* convey a dynamic binding pocket. *A*, significant changes in structure result from the reduction or oxidation of the cofactor in the absence of substrate. These rearrangements might allosterically promote the uptake of tryptophan for product formation by inducing a more dynamic binding pocket. *B*, the presence of substrate allosterically influences the structure of the cofactor binding site. Reduction or oxidation of the cofactor only causes small changes in protein structure because all requirements for product formation are already fulfilled. *C*, in the absence of any halide, the cofactor-binding site becomes more polar and flexible, which almost eliminates the allosteric coupling to the substrate-binding site.

Changes in secondary structure are the most pronounced in the presence of chloride and the absence of tryptophan and are minimal in the presence of tryptophan. We suggest that these responses might constitute a mechanism for the promotion of the enzymatic reaction (Fig. 6). All observations were obtained by reduction of the FAD_ox_ but will accordingly inversely accompany the oxidation of FADH^−^ during the formation of HOCl. The large signals in the absence of tryptophan imply a regulation mechanism of the enzyme, which might promote the binding of tryptophan after HOCl has been formed and is guided to the empty substrate-binding site. In contrast, only small changes in the protein structure take place if all substrates including tryptophan are already present and the cofactor is oxidized. The diffusion of the intermediary HOCl from the FAD-binding pocket to the site of catalysis does not require any extensive protein rearrangements (21). The structural changes are more pronounced in the presence of the product 5-chlorotryptophan than in the presence of tryptophan. An opening or higher flexibility of the structure might therefore also constitute part of a release mechanism of the product. When we added bromide instead of chloride, the changes in secondary structure became less pronounced. The presence of bromide might lead to a less rigid structure and therefore reduce the coupling of both binding pockets. The result would reduce tryptophan binding to the enzyme and therefore disfavor the reaction with bromide. Such an effect might contribute to the observed reduction in the conversion of substrate to 75% of the conversion in the reaction with chloride (14).

In the absence of halide, we observed very little changes in secondary structure. In this regard, the difference spectrum might show some similarities to that in the presence of chloride and tryptophan. However, without halide present, the enzyme loses most of its capacity to induce allosteric feedback responses. As the flavin-binding pocket becomes more flexible and polar by loss of halide, some interactions necessary for the regulation of enzyme activity might no longer be active.

## Conclusions

We demonstrate the allosteric response in α-helices and β-sheet of tryptophan 5-halogenases to changes in the redox state of the FAD. Oxidation of FAD accompanies the formation of hypohalous acid as fundamental step in the halogenase mechanism, which is accordingly coupled via structural changes to the substrate-binding site. The advantage of the spectroscopic approach is that many different conditions could be systematically studied including product binding and the complete absence of halide. Moreover, the interconversion of two functional states was observed. These results complement previous fundamental investigations by crystallography and kinetic studies and emphasize the communication between the cofactor and substrate-binding sites despite their clear spatial separation. In addition, the significant structural response to photoreduction of FAD_ox_ can be used as an indicator of functional and detrimental variants with respect to allostery.

One question that has not yet been clarified with this method is the influence of oxygen on the reaction. All samples measured here were anaerobic due to incubation with the high DTT concentration in the sample and the photoreduction of flavin. Therefore, signals accompanying the oxidation of FADH^−^ and formation of hypohalous acid could not be investigated directly. This question might be addressed in the future by supplying oxygen to the sample via photorelease from a caged compound (54), which would also facilitate an investigation of further reaction steps leading to the formation of halogenated product.

## Experimental procedures

All buffers were prepared using ultrapure water (Arium Pro VF, Sartorius) with chemicals purchased from VWR or Carl Roth if not indicated otherwise.

### Protein expression, purification, and sample preparation

PyrH (amino acids 1–511) (14) with N-terminal 6× His-tag was expressed in *Escherichia coli* BL21 (DE3) pLysE and purified via affinity chromatography as described previously (22). The purification buffer was exchanged to the reconstitution buffer by using a filter device (Amicon Ultra 4, Merck) with a 50 kDa cutoff. The sample was incubated in the reconstitution buffer with 1 mM FAD (>95% for biochemistry, Carl Roth) overnight at 4 °C. Samples were obtained in the final buffer after three additional washing steps each with a dilution of more than 1:10, which reduced the concentration of free FAD in solution to <1% of that of bound FAD.

For samples with chloride, the reconstitution buffer contained 50 mM sodium phosphate, pH 8.3, 1 mM FAD, 20% (v/v) glycerol (>99.5%, Thermo Fisher Scientific), and 300 mM NaCl, whereas the final buffer was 60 mM potassium phosphate, pH 8.3, 1% (v/v) glycerol, and 20 mM NaCl. For samples with bromide, the reconstitution buffer contained 60 mM sodium phosphate, pH 8.3, 1 mM FAD, 20% (v/v) glycerol, and 300 mM NaBr (99.99%, Thermo Fisher Scientific), whereas the final buffer was 60 mM potassium phosphate, pH 8.3, 1% (v/v) glycerol, and 20 mM NaBr. For samples without halide, the reconstitution buffer contained 60 mM sodium phosphate, pH 8.3, 1 mM FAD, 20% (v/v) glycerol, whereas the final buffer was 60 mM potassium phosphate, pH 8.3, and 1% (v/v) glycerol. All samples for FTIR experiments were concentrated to an A_450 nm_ >10, frozen in liquid nitrogen, and stored at −80 °C.

### Isotope labeling

u-^13^C-PyrH was obtained by expression in *E. coli* BL21 (DE3) pLysE as described above but in M9 minimal medium with u-^13^C_6_ glucose (99%, Eurisotop, 8 g/L). Purification and preparation were performed as with the protein at natural isotope abundance. The reconstitution buffer contained 50 mM sodium phosphate, pH 8.3, 1 mM FAD, 20% (v/v) glycerol, and 300 mM NaCl, whereas the final buffer was 60 mM potassium phosphate, pH 8.3, 1% (v/v) glycerol, and 20 mM NaCl. The samples were concentrated to an A_450 nm_ > 10, frozen in liquid nitrogen, and stored at −80 °C.

### UV-vis spectroscopy

UV-vis spectra were recorded immediately after the buffer exchange. Spectra were recorded in a quartz cuvette (Suprasil, Hellma) with a 1 cm path length using a Shimadzu UV-2450 spectrometer. The spectral resolution was set to 0.5 nm. The occupancy of PyrH with FAD was determined using an extinction coefficient for PyrH of ε_280_ = 99,155 L mol^−1^ cm^−1^ calculated using ProtParam (https://web.expasy.org/protparam) and the extinction coefficients for free FAD of ε_450_ = 11,300 L mol^−1^ cm^−1^ (49) and ε_280_ = 19,312 L mol^−1^ cm^−1^ as derived from a UV-vis spectrum of FAD.

### FTIR difference spectroscopy

To 12 μl of protein sample, 1 μl of 500 mM DTT (99.5%, AppliChem) in 60 mM potassium phosphate buffer, pH 8.3, was added to a final concentration of 35 mM. For experiments in the presence of tryptophan or 5-chloro-tryptophan, additionally 1 μl of 40 mM tryptophan (Sigma-Aldrich) or 5-chloro-tryptophan (Santa Cruz Biotechnology) was added to the sample to a final concentration of 3 mM. The sample was stored for at least 24 h on ice in the dark to achieve mixing and a binding equilibrium. 1.6 μl of the sample were placed on a BaF_2_ window (20 mm diameter) with vacuum grease on the edge and immediately sealed with a second window without any drying. The path length of the sandwich cuvette of below 10 μm was adjusted to result in an absorption of protein and water of *A* < 1.0 at 1650 cm^−1^. The sealed cuvette was kept in the spectrometer for at least 2 h to equilibrate the sample and to create a low oxygen concentration by the presence of 35 mM DTT.

FTIR spectra were recorded on a Bruker IFS 66v/s spectrometer equipped with a mercury cadmium telluride detector with a spectral resolution of 2 cm^−1^ at 10 °C. FT was performed with a zero filling of four. Difference spectra were obtained using a long wave pass filter to restrict the measurement range to below 2256 cm^−1^. Spectra with 1024 scans were recorded until a stable baseline was obtained. Then, the sample was illuminated for 5 to 10 min with a blue LED at 450 nm (∼22 mW/cm^2^, Philips Lumileds) until a maximal signal intensity was reached. Measurements for up to 30 min after illumination were recorded in intervals of 1024 scans and those with a stable baseline were selected. Several independent preparations were investigated and finally averaged to 44,032 scans for PyrH with chloride, 50,176 scans for PyrH with bromide, 38,912 scans for PyrH without halide, 9216 scans for PyrH with tryptophan and chloride, 24,576 scans for PyrH with 5-chloro-tryptophan and chloride, and 12,288 scans for ^13^C-labeled PyrH.

### Determination of water content in samples for FTIR spectroscopy

To analyze the hydration of the halogenase, 23 μl of a representative sample of PyrH for FTIR spectroscopy with a concentration of 1 mM was placed in a glass vial. The vial was weighed with a precision balance (Precisa LS 320A SCS). The sample was flash frozen using liquid nitrogen and freeze-dried for 72 h using an Alpha 2-4 LSCbasic lyophilizer (Martin Christ). Afterward, the vial was weighed again and the amount of evaporated water was calculated to 0.0166 g. Using the molar mass of PyrH of 58.7 kDa, the ratio was calculated to 13 g of water per 1 g of protein, which corresponds to a high sample hydration of about 40,000 water molecules per protein.

## Data availability

All data are contained in the manuscript and the supporting information.

## Supporting information

This article contains supporting information (55, 56).

## Conflict of interest

The authors declare that they have no conflicts of interest with the contents of this article.
