# Supplementary material for: Coupling and regulation mechanisms of the flavin-dependent halogenase PyrH observed by infrared difference spectroscopy
Source: J Biol Chem. 2024 Mar 20;300(4):107210. doi: 10.1016/j.jbc.2024.107210 (PMC11021962; doi:10.1016/j.jbc.2024.107210)
Supplement: Supporting Figures S1–S7 and Table S1 [file mmc1.pdf]

Supporting Information for

Coupling and regulation mechanisms of the flavin-dependent halogenase PyrH observed by infrared difference spectroscopy

Lea Schroeder[1], Niklas Diepold[1,2], Simon Gäfe[1], Hartmut H. Niemann[3], Tilman Kottke[1,2]

[1]Biophysical Chemistry and Diagnostics, Department of Chemistry, Bielefeld University, Universitätsstraße 25, 33615 Bielefeld, Germany

[2]Biophysical Chemistry and Diagnostics, Medical School OWL, Bielefeld University, Universitätsstraße 25, 33615 Bielefeld, Germany

[3]Structural Biochemistry, Department of Chemistry, Bielefeld University, Universitätsstraße 25, 33615 Bielefeld, Germany

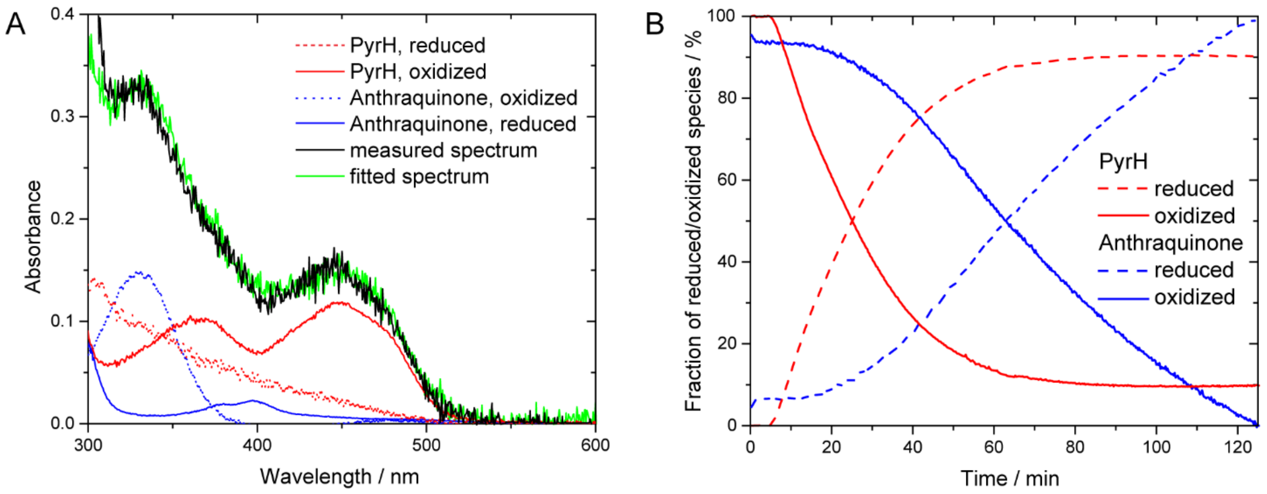

Figure S1: The redox potential of FAD in PyrH was determined using the xanthine / xanthine oxidase system in conjunction with a reference dye as has been shown for other heme and FAD enzymes (1,2). PyrH (20 µM in 50 mM Tris-HCl, pH 8.0) was analyzed in a solution comprising xanthine (1.5 mL, saturated), glucose (200 µL, 100 mM), EDTA (200 µL, 10 mM), methyl viologen (10 µL, 400 µM), xanthine oxidase (5 µL, 20 mg/mL), glucose oxidase (20 µL, 5 mg/mL) and the reference dye 2-anthraquinone-sulfonate (35 µL, 2 mM). The solutions were purged with argon during the whole process and kept at 20 °C. Spectra were recorded in 15 s intervals and analyzed by a script written in Matlab R2012b (The Mathworks) using a least squares fit of reference spectra of each component. An example of the fitting procedure at the time point of 26 min is shown in (A), which was used to obtain the ratio of reduced and oxidized species at each time point accordingly, as shown in (B).

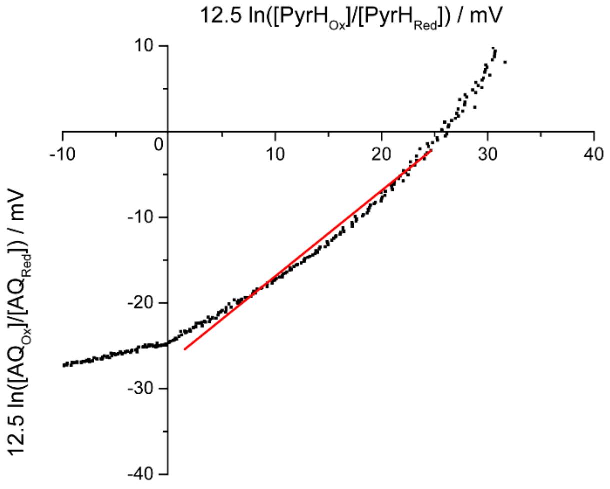

Figure S2: Nernst plot for determination of the redox potential from the ratios of oxidized and reduced species in Fig. S1 (2). The redox potential was obtained from the y-axis intercept of the linear fit with a given slope of one (shown in red). The redox potential of the reference dye 2-antraquinone-sulfonate of E_0' = -225 mV vs. SHE at 20 °C was used (1) and corrected for pH 8.0 to E = -277 mV vs. SHE (3). Accordingly, the fit yields a redox potential of PyrH at pH 8 of E = -248 ± 4 mV vs. SHE from four separate experiments.

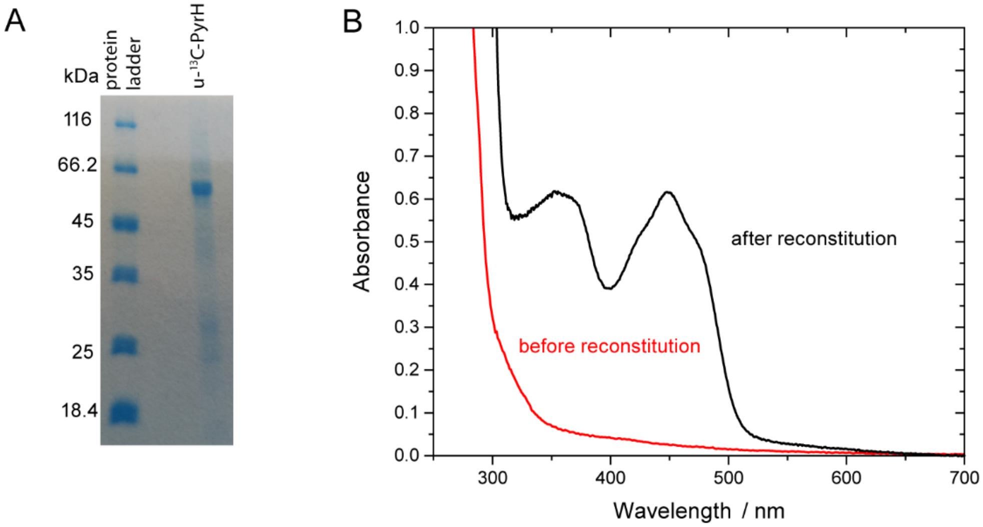

Figure S3: Purification and reconstitution of u-^{13}C-PyrH. (A) An SDS-PAGE of purified u-^{13}C-PyrH is shown in comparison to a protein ladder (Pierce Unstained Protein Molecular Weight Marker SM26610). The molecular weight of the purified protein agrees with the theoretical weight of 58 kDa. (B) UV-vis spectra of u-^{13}C-PyrH before and after reconstitution were recorded. Without reconstitution (red) an absorbance of FAD was not detected, whereas after reconstitution (black) the typical spectrum of FAD bound to the protein was obtained. Accordingly, all FAD in u-^{13}C-PyrH is at natural abundance of isotopes, because the FAD was incorporated to the protein only during reconstitution.

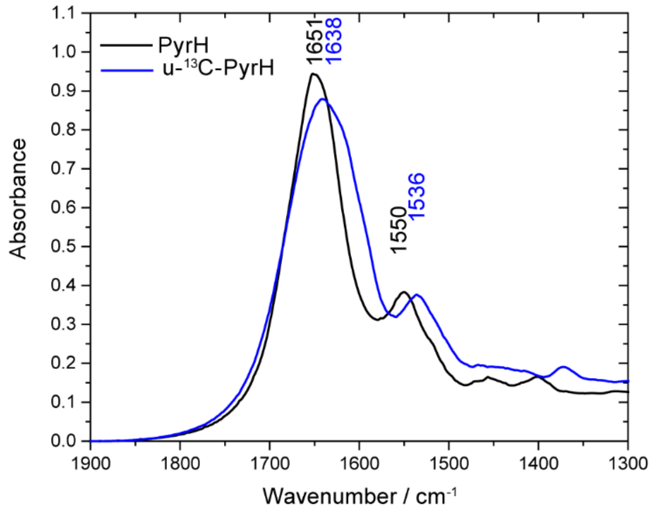

Figure S4: FT-IR spectra of u-^{13}C-PyrH (blue) in comparison to PyrH at natural isotope abundance (black). A downshift in the amide I band by 13 cm^{-1} and in the amide II band by 14 cm^{-1} were observed in agreement with band positions of other u-^{13}C-labeled proteins (4).

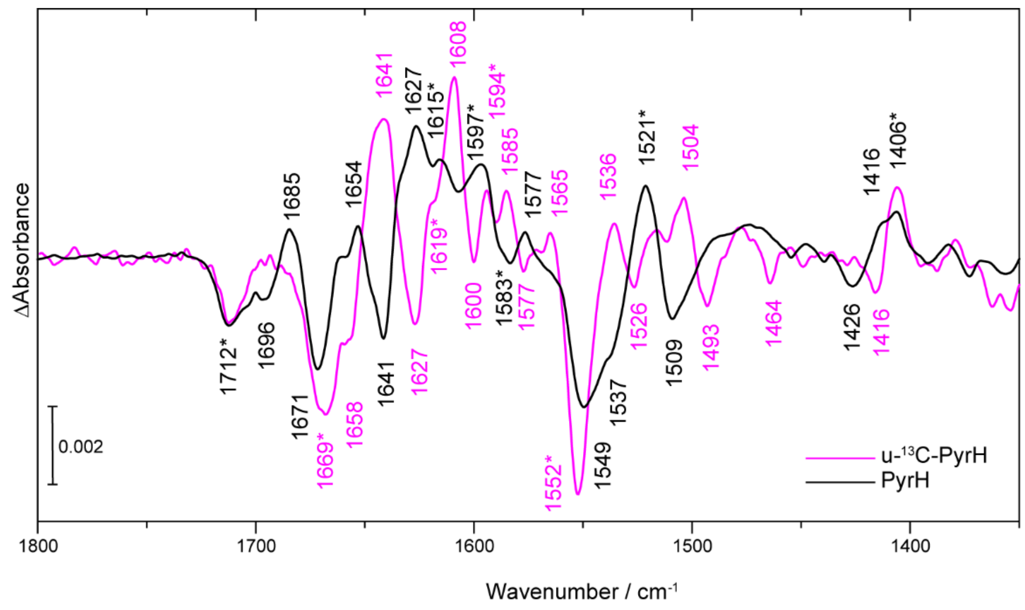

Figure S5: FT-IR difference spectra of the reduction of FAD at natural isotope abundance in u-^{13}C-PyrH (magenta) and PyrH (black) covering the whole spectral region. Bands marked with an asterisk do not shift and therefore originate from FAD. Small differences in wavenumber of FAD bands between both samples are caused by an overlap with bands of the protein moiety.

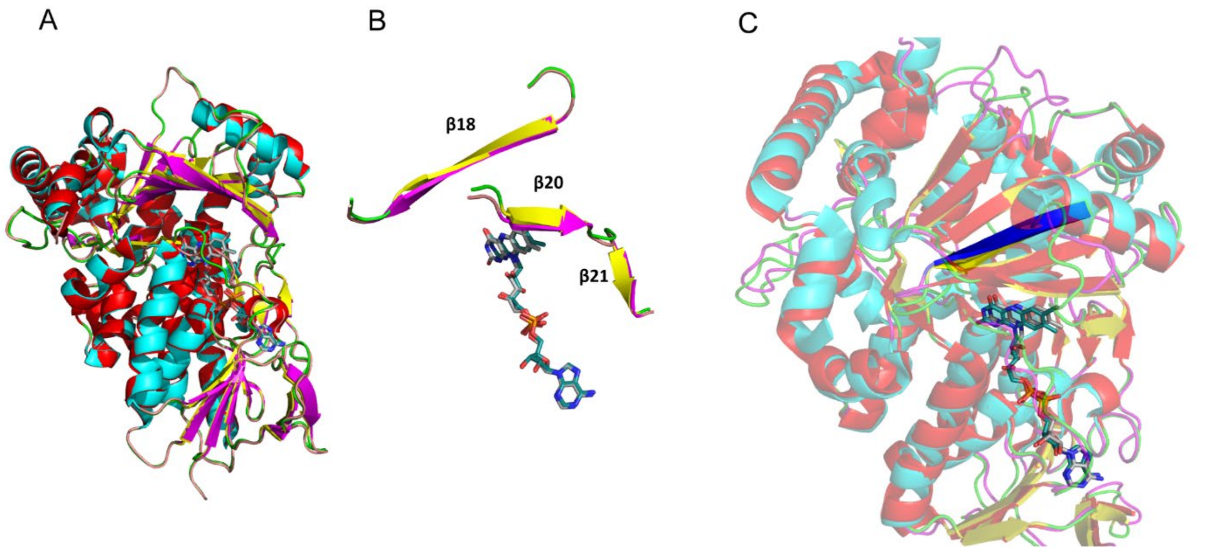

Figure S6: Comparison of crystal structures of PrnA with bound FAD_{ox} and $FADH^{-}$, respectively. (A) Overlay of α-helices (red) and β-sheets (yellow) for PrnA with FAD_{ox} and α-helices (cyan) and β-sheets (magenta) for PrnA with $FADH^{-}$, respectively (PDB entry 2APG and 2ARD) (5). (B) View on a selected β-sheet element shows an elongation of β18 depending on the redox state of FAD. This elongation was confirmed by analysis of secondary structure using the DSSP algorithm (6) (https://www3.cmbi.umcn.nl/xssp/). (C) The overlay of the structures of PrnA with bound FAD_{ox} and PyrH with bound FAD_{ox} demonstrates that the β-sheet element β18 (highlighted in blue) is conserved in position and structure in PyrH (PDB entry 2WET) (7).

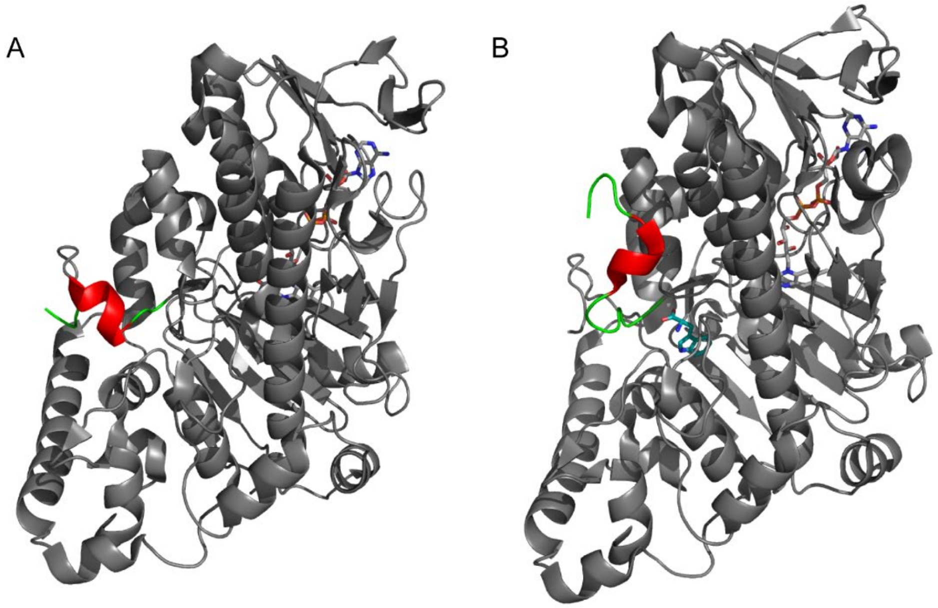

Figure S7: Crystal structures of PyrH with FAD_{ox} in the absence and the presence of tryptophan. (A) In the absence of tryptophan, the binding loop is open and mostly dynamic, but some residual part of the loop forms an α-helical secondary structure. (B) In the presence of tryptophan, the binding loop is closed and partially forms an α-helical element, which differs from that in the absence of tryptophan (PDB entry 2WET) (7).

Table S1: Assignment of flavin bands in PyrH based on isotope labeling and comparison to the difference spectrum of reduced *minus* oxidized flavin mononucleotide ($FMNH^-$ / FMN_{ox}) in solution (8).

Band position in cm^{-1} of PyrH		Band position in cm^{-1} of		Assignment
neg.	pos.	FMN_{ox}	$FMNH^-$	
1712		1712		ν C_4=O (8)
1669		1661		ν C_2=O, δ N_3H (8)
	1615		1631	ν C=O
	1597		1601	ν C_{4a}=C_{10a}
1583		1581		ν CN ring (II), ν CC ring (I), ν CN ring (III) (8)
1552		1549		ν CC ring (I), ν CN ring (II) (8)
	1521		1517	
	1406		1411	

Supporting References

1. Arents, J. C., Perez, M. A., Hendriks, J., and Hellingwerf, K. J. (2011) On the midpoint potential of the FAD chromophore in a BLUF-domain containing photoreceptor protein. *FEBS Lett.* **585**, 167-172
2. Efimov, I., Parkin, G., Millett, E. S., Glenday, J., Chan, C. K., Weedon, H., Randhawa, H., Basran, J., and Raven, E. L. (2014) A simple method for the determination of reduction potentials in heme proteins. *FEBS Lett.* **588**, 701-704
3. Conant, J. B., Kahn, H. M., Fieser, L. F., and Kurtz, S. S. (1922) An electrochemical study of the reversible reduction of organic compounds. *J. Am. Chem. Soc.* **44**, 1382-1396
4. Sommer, C., Dietz, M. S., Patschkowski, T., Mathes, T., and Kottke, T. (2017) Light-induced conformational changes in the plant cryptochrome photolyase homology region resolved by selective isotope labeling and infrared spectroscopy. *Photochem. Photobiol.* **93**, 881-887
5. Dong, C., Flecks, S., Unversucht, S., Haupt, C., van Pée, K. H., and Naismith, J. H. (2005) Tryptophan 7-halogenase (PrnA) structure suggests a mechanism for regioselective chlorination. *Science* **309**, 2216-2219
6. Kabsch, W., and Sander, C. (1983) Dictionary of protein secondary structure: pattern recognition of hydrogen-bonded and geometrical features. *Biopolymers* **22**, 2577-2637
7. Zhu, X., De Laurentis, W., Leang, K., Herrmann, J., Ihlefeld, K., van Pée, K. H., and Naismith, J. H. (2009) Structural insights into regioselectivity in the enzymatic chlorination of tryptophan. *J. Mol. Biol.* **391**, 74-85
8. Thöing, C., Pfeifer, A., Kakorin, S., and Kottke, T. (2013) Protonated triplet-excited flavin resolved by step-scan FTIR spectroscopy: implications for photosensory LOV domains. *Phys. Chem. Chem. Phys.* **15**, 5916-5926
